# Dynamic variable selection in SNP genotype autocalling from APEX microarray data

**DOI:** 10.1186/1471-2105-7-521

**Published:** 2006-11-30

**Authors:** Mohua Podder, William J Welch, Ruben H Zamar, Scott J Tebbutt

**Affiliations:** 1 Department of Statistics, University of British Columbia, Vancouver, BC, Canada; 2 James Hogg iCAPTURE Centre for Cardiovascular and Pulmonary Research, St. Paul's Hospital, Vancouver, BC, Canada

## Abstract

**Background:**

Single nucleotide polymorphisms (SNPs) are DNA sequence variations, occurring when a single nucleotide – adenine (A), thymine (T), cytosine (C) or guanine (G) – is altered. Arguably, SNPs account for more than 90% of human genetic variation. Our laboratory has developed a highly redundant SNP genotyping assay consisting of multiple probes with signals from multiple channels for a single SNP, based on arrayed primer extension (APEX). This mini-sequencing method is a powerful combination of a highly parallel microarray with distinctive Sanger-based dideoxy terminator sequencing chemistry. Using this microarray platform, our current genotype calling system (known as SNP Chart) is capable of calling single SNP genotypes by manual inspection of the APEX data, which is time-consuming and exposed to user subjectivity bias.

**Results:**

Using a set of 32 Coriell DNA samples plus three negative PCR controls as a training data set, we have developed a fully-automated genotyping algorithm based on simple linear discriminant analysis (LDA) using dynamic variable selection. The algorithm combines separate analyses based on the multiple probe sets to give a final posterior probability for each candidate genotype. We have tested our algorithm on a completely independent data set of 270 DNA samples, with validated genotypes, from patients admitted to the intensive care unit (ICU) of St. Paul's Hospital (plus one negative PCR control sample). Our method achieves a concordance rate of 98.9% with a 99.6% call rate for a set of 96 SNPs. By adjusting the threshold value for the final posterior probability of the called genotype, the call rate reduces to 94.9% with a higher concordance rate of 99.6%. We also reversed the two independent data sets in their training and testing roles, achieving a concordance rate up to 99.8%.

**Conclusion:**

The strength of this APEX chemistry-based platform is its unique redundancy having multiple probes for a single SNP. Our model-based genotype calling algorithm captures the redundancy in the system considering all the underlying probe features of a particular SNP, automatically down-weighting any 'bad data' corresponding to image artifacts on the microarray slide or failure of a specific chemistry. In this regard, our method is able to automatically select the probes which work well and reduce the effect of other so-called bad performing probes in a sample-specific manner, for any number of SNPs.

## Background

### Genotyping SNPs

Determination of the alleles at a specific single nucleotide polymorphism (SNP) site is called genotyping. An optimal genotyping technology should be capable of genotyping any number of SNPs for a large number of individuals satisfying the following criteria: 1. easy and quick development of an assay from the sequence information; 2. over-all low cost; 3. the data analysis must be simple, transparent, fully-automated and robustly give accurate genotype-calls for all kinds of samples; and 4. the study design must be flexible and scalable in all respects (e.g., number of SNPs investigated). Automated genotype calling is an essential part of such a system. A number of medium to high-throughput genotyping methods have been developed. Among these various techniques, TaqMan [1] was designed optimally to give genotypes of large numbers of individuals for one SNP at a time. But from a clinically relevant, personalized medicine point of view, we require a system which can genotype multiple SNPs simultaneously for any single patient sample.

Such a system can be achieved through a device known as a genotyping microarray. Through this mechanism, one can display thousands of specific oligonucleotide probes, precisely located on a small glass slide. These array-based technologies offer both economic and patient specific applications allowing the genotyping of multiple SNPs simultaneously. There are a number of microarray genotyping protocols, including Affymetrix GeneChips^(*R*) ^[2] and Illumina's BeadArray™ system [3]. For the widely used Affymetrix GeneChip system, a system based on the discriminatory power of nucleic acid hybridization to generate the genotyping signals, sophisticated autocalling algorithms have been developed [4]. Over the last five to six years Affymetrix has developed and tested a series of algorithms using their platform. The Affymetrix GeneChip is suitable for very large scale genotyping, e.g., 10,000 or more SNPs at a time, but is expensive for medium to small scale genotyping (e.g., 100 to 200 SNPs). The Illumina BeadArray genotyping platform provides a powerful combination of high-throughput and accuracy with low cost per SNP analysis. Based on the GoldenGate™ genotyping assay, Illumina designed a genotype calling algorithm using a Bayesian model, taking the ratio of two single colored intensity signals corresponding to two possible SNP alleles, to give the genotype for a single SNP [5]. The automatic calling of genotypes is performed by proprietary software, GenCall, which is based on a custom-designed clustering algorithm [5]. To our knowledge, exact details of the algorithm are not available in the public domain.

Compared to these systems, our laboratory has developed a robust and redundant chemistry platform using the technology of single base extension which produces multiple signals from multiple probes [APEX and allele-specific APEX (ASO) probes for both DNA strands] corresponding to a single SNP [6]. To our knowledge, APEX is the only chemistry in which the on-chip assay can be performed in 20 minutes, making APEX potentially suitable for rapid genetic diagnostics in clinical settings: the Affymetrix assay takes several hours for hybridization on the chip, and Illumina's assays also takes longer compared to APEX.

Commercial software called Genorama [7] can detect all the four colors of fluorescence emitted from the dyes used in an APEX experiment, and then automatically call the base(s) corresponding to a specific probe spot. The problem with this system is that the underlying scoring algorithm treats all probes equally and thus requires considerable inspection of the original array data to produce the final genotype call [8,9]. Using the Genorama base-calling data for both APEX and AS-APEX probes, Gemignani et al. [10] developed a simple matrix-score based algorithm and made the calls corresponding to the most likely genotype, but with considerable manual inspection.

### Current Genotype Calling System: SNP Chart

**Figure 1:**
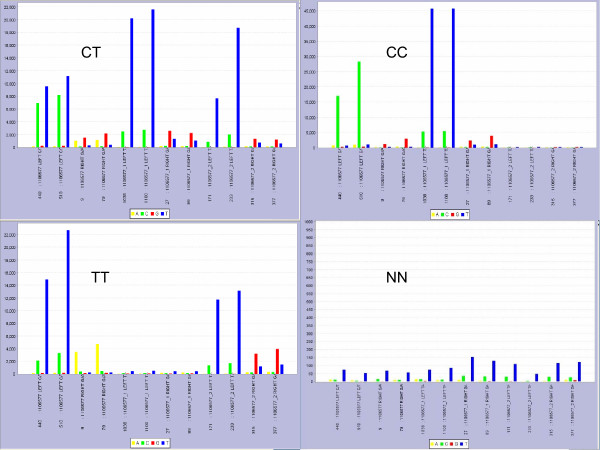
**Examples of SNP Chart Application**. Examples of SNP Charts for the SNP rs1106577 to illustrate the data structure (e.g., Table 1). Template DNA from three Coriell samples with three possible genotypes (CC, CT and TT) and one negative control (NN) are shown in four different charts. Each chart shows four-channel fluorescent intensity data (A, C, G, and T) on the vertical axes, from 12 rs1106577-specific array spots (duplicate spots for six different probes). On the horizontal axes, 12 probe-names corresponding to 12 spots are given sequentially. 1st and 2nd spots from the left ("LEFT C/T") refer to the left-hand APEX probe that will give either a single C (green) signal (for homozygous CC genotypes) or a T (blue) signal (for homozygous TT genotypes) or a mixture of C and T (heterozygous CT). 3rd and 4th spots from the left ("RIGHT G/A") refer to the right-hand APEX probe that interrogates the DNA strand nucleotide complementary to that of the left-hand APEX probe, thus giving a single G (red) signal (for CC), a single A (yellow) signal (for TT), or a mixed G and A signal (for CT). From left, spots 5 to 12, inclusive, represent allele-specific APEX probes in which a base-specific fluorescence signifies the presence of the allele. Among them, spots 5 to 8 refer to the "_1" probes corresponding to the first allele (C in the case of rs1106577) and spots 9 to 12 refer to the "_2" probes corresponding to the second allele (T). The redundancy and consistency of the data across different probes give high confidence in the assigned genotypes.

SNP Chart is a Java based visualization tool, developed by our research group [11]. In this integrated platform, spot intensity data from different and/or replicate probes (randomly scattered across the microarray slide) that interrogate the same SNP are imported, together with a multi-channel TIFF image of the original array experiment. This system is capable of calling any SNP genotype with the help of individual manual data inspection. The main problem with this genotype-calling system is that it is time-consuming and exposed to user subjectivity bias. Examples of SNP Charts are shown in Figure 1.

**Table 1:** Data structure for SNP rs1106577 and DNA sample Coriell NA17102 (CC) (CC-chart in Figure 1)

*Spot ID*	*Probe ID*	*Expected allele ID*	A	C	G	T
Spot 1	APEX_LEFT	C and/or T	732	**17003**	258	**667**
Spot 2	APEX_LEFT	C and/or T	965	**28290**	348	**1046**
Spot 3	APEX_RIGHT	G and/or A	**190**	85	**1198**	233
Spot 4	APEX_RIGHT	G and/or A	**353**	104	**2923**	269
Spot 5	ASO_1LEFT	T	109	5284	80	**45700**
Spot 6	ASO_1LEFT	T	107	5456	83	**45713**
Spot 7	ASO_2LEFT	T	90	88	20	**182**
Spot 8	ASO_2LEFT	T	76	106	22	**222**
Spot 9	ASO_1RIGHT	G	288	182	**2346**	992
Spot 10	ASO_1RIGHT	G	369	209	**3908**	1098
Spot 11	ASO_2RIGHT	G	138	68	**166**	187
Spot 12	ASO_2RIGHT	G	151	68	**212**	193

### Data Composition

We built our genotyping model based on the training set of 32 Coriell DNA samples [12] and 3 negative PCR controls [6,11]. Each sample comes from a single microarray experiment, conducted on a small glass slide, and contains information on all the SNPs under study. Our laboratory has developed a robust microarray platform for each sample patient, generating multiple signals for approximately one hundred SNPs using two kinds of probes, namely, classical APEX probes and allele specific APEX (ASO) probes [6]. There are six probes in total for each biallelic SNP and each probe has two replicates which make twelve different spots for a single SNP on the microarray slide. All these spots are randomly scattered across the microarray slide but with known coordinates. Multiple sets of probes of these types along with their replicates make this genotyping platform unique and redundant. Each spot in the microarray slide produces signals from four different channels, corresponding to A, C, G and T. In our current genotyping method, we only considered the expected foreground signals and will consider all the background, non-expected signals for further development of genotyping model (see below).

An example of a data source for a single Coriell sample and a single SNP is given in Table 1. For the SNP rs1106577, the two possible alleles are C or T. Each row of this table represents a single spot. The first column is the spot ID; the second column is the probe name; the third column is the expected allele ID for the appropriate spot; and the last four columns are the signal intensity values for the four channels corresponding to each spot.

In the second column of Table 1, "APEX_LEFT" refers to the left-hand APEX probe on the sense strand, and "APEX_RIGHT" refers to the right-hand APEX probe on the anti-sense strand that interrogates the DNA strand nucleotide complementary to that of the left-hand APEX probe. For all the APEX probes, the fluorescent signals come from the base position of the SNP allele. In contrast, for all the ASO probes, fluorescent signals come from the base adjacent (3') to the actual SNP site [6]. For the SNP rs1106577 considered in Table 1, the base 3' adjacent to the SNP allele is always T on the sense strand. The left probes, ASO_1LEFT and ASO_2LEFT, are designed to signal at this adjacent base, T, if the SNP site has the first allele (C here) and/or the second allele (T here), respectively. Similarly, SNP rs1106577 has G in the adjacent position 3' to the SNP side on the anti-sense strand. The right probes, ASO_1RIGHT and ASO_2RIGHT, signal at this adjacent base, G, again for C and/or T at the SNP site, respectively. It is merely the presence or absence of the signal that indicates the SNP allele. According to the probe structure, the signals corresponding to the expected alleles are highlighted.

The data represented in Table 1 come from the DNA sample Coriell NA17102 and here the true genotype is CC (see the top-right CC-chart in Figure 1). According to the APEX chemistry, for the genotype CC the dominating signals from spots 1 and 2 should be C among the two expected channels C and T. Similarly the dominating signals from spots 3 and 4 should be G (complementary to C in the left-strand) among the two expected channels G and A. Rows 5–12 represent the ASO probes in which a base-specific fluorescence signifies the presence of the allele. Since the genotype is CC, all the expected signals corresponding to allele 1 (C) should dominate over the other channels, i.e., expected foreground (expected channel corresponding to all allele 2 probes) and background signals [6]. Note that for spots 11 and 12, the expected signal (G), corresponding to the presence of the T allele (which is absent in this particular case), is comparable to the background signals. In Table 1, all the signals which are not highlighted in bold are considered as background signals, often due to the spectral overlap between dyes, and/or a general background.

In fact, this is a very good source of data, as all the signals corresponding to allele 2 (T in this case) are comparable to the level of background signals. Now suppose the true genotype is TT, then we should expect dominating signals only from the expected channels corresponding to all allele 2 probes. For a heterozygous CT genotype, we should expect dominating signals from all the expected channels corresponding to both allele 1 probes and allele 2 probes. These features of our redundant and robust platform can also be represented through our data visualization tool: SNP Chart [11]. In Figure 1, four SNP Charts corresponding to three different genotypes (CT, CC and TT) and a negative control (NN) are shown for the same SNP (rs1106577).

In our study we use the 32 Coriell samples plus three negative PCR controls for model building. These 35 samples will be called the Coriell training set. To test the performance of the calling algorithm we also have a completely independent set of 270 SIRS (systematic inflammatory response syndrome) DNA samples from the ICU of St. Paul's hospital, plus one test negative control sample. This set of 271 samples will be called the SIRS test data. Note that the SIRS data are not used in model building and come from a separate study, so they provide a very rigorous test. For the training data, there are 123 SNPs on the microarray slide, but only 96 were usable: (1) 15 SNPs had PCR chemistry failure and (2) 12 SNPs had one of the three possible genotypes missing among the training set.

### Formation of Classifiers

Ideally, the genotype call could be solely based on just one of four sets of probes: (1) APEX_LEFT, (2) APEX_RIGHT, (3) ASO_1LEFT and ASO_2LEFT, and (4) ASO_1RIGHT and ASO_2RIGHT (see Table 1). Accordingly, we have developed four sets of classifiers, named APEX.L, APEX.R, ASO.L and ASO.R, based on the respective probe sets. Each classifier is based on two explanatory variables, generically denoted by X and Y, measuring the signal intensities for the two candidate alleles in the SNP position. In Table 1, for example, X and Y corresponds to the C and T alleles, respectively.

Between them the four classifiers have four pairs of such explanatory variables: (APEX.XL, APEX.YL); (APEX.XR, APEX.YR); (ASO.XL, ASO.YL) and (ASO.XR, ASO.YR). They are derived from the signal intensities in rows 1–2, 3–4, 5–8, and 9–12, respectively, in data structures exemplified in Table 1. All these variables take the sum of the relevant signals. From the example data in Table 1, the values of the variables for the classifier APEX.L are APEX.XL = 17, 003 + 28, 290 = 45, 293 and APEX.YL = 667 + 1, 046 = 1, 713, and so on, as summarized in Table 2.

**Table 2:** Values of the explanatory variables for SNP rs1106577 and DNA sample Coriell NA17102

*Classifier*	*Variables used by classifier*		*Values*	
APEX.L	APEX.XL	APEX.YL	45,293	1,713
APEX.R	APEX.XR	APEX.YR	4,121	543
ASO.L	ASO.XL	ASO.YL	91,413	404
ASO.R	ASO.XR	ASO.YR	6,254	378

Our main objective is to automatically select from these four sets of variables those pairs which give "good" signals for genotype calling. Moreover, the variables and hence the classifier(s) used will be chosen dynamically, i.e., for a specific SNP and sample. In this paper we use Fisher's [13] linear discriminant analysis (LDA) to build the classifiers, but the method of dynamic variable selection would apply to any linear or nonlinear classifier.

**Figure 2:**
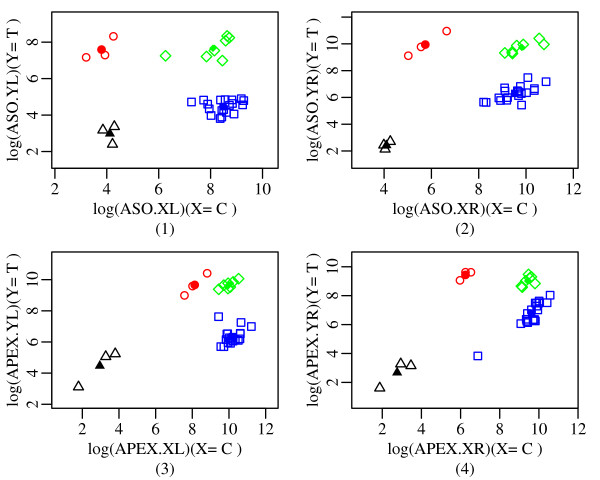
**Example of a well-behaved SNP: rs1932819**. All the classifiers give three well separated clusters for the SNP rs1932819 [red, green, blue and black colored symbols respectively denote the classes YY, XY, XX and NN].

**Figure 3:**
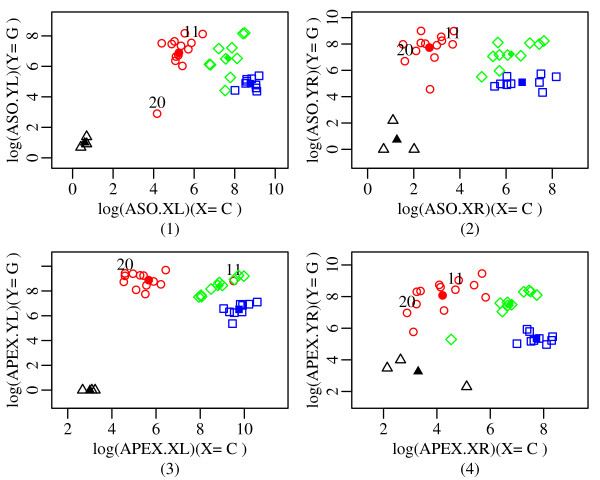
**Example of critical SNP: rs1003399**. Sample 11 is correctly classified by both ASO probes and APEX.R probe but wrongly classified by APEX.L probe for the SNP: rs1003399, whereas for sample 20, APEX.L probe works the best [red, green, blue and black colored symbols respectively denote the classes YY, XY, XX and NN].

Figure 2 and Figure 3 illustrate how dynamic variable selection exploits the redundancy in the chemistry. The figures are based on the 32 Coriell samples plus three negative PCR controls, where the true genotypes are known. We plot the X and Y signals for each of the four probe sets. Ideally, any pair of variables would form well separated clusters for the three possible genotypes, XX, XY and YY (plotted with different colors and symbols). There is a fourth cluster corresponding to the negative controls (NN). Any reasonable classifier based on these variables should make correct calls under ideal conditions. Figure 2 shows an ideal SNP, where all four probe sets produce good separation of the three genotypes and the negative controls.

Conversely, problems with the samples or the chemistry may lead to overlap in the four clusters, making calling difficult. In Figure 3 for SNP rs1003399, for example, sample 11 is a GG genotype which falls in the CG cluster for the left APEX probe set. Fortunately, the other three probe sets correctly place sample 11 in the GG cluster. So three out of the four probe sets work, and classifiers based on them would make the correct call for sample 11. For sample 20 (NA07341), however, the left APEX probe set works the best, placing the GG sample clearly in the GG data cluster. Thus, different probe sets may be effective for different samples, even for the same SNP. Our algorithm attempts to identify effective probe sets automatically, sample by sample, and it is in this sense that it chooses variables dynamically.

## Results and Discussion

### Dynamic-variable LDA Based Genotyping Model

For each SNP we build four separate LDA classification models; the models are based on the pairs of explanatory variables in Table 2 corresponding to the four probe sets. For this stage the training data are the 32 Coriell samples and the three negative PCR controls described under Data Composition. As test data to evaluate the calling performance we use the 271 SIRS test samples also described under Data Composition. Within each SNP, sample by sample the four classifiers are combined using the weighting algorithm described later in the Methods Section, to give one call for the particular test sample. The calls are checked for concordance with the validated genotypes in the SIRS data, leading to the results in the first row of Table 3. In 0.4% of samples, the called genotype is NN (non-call), hence the call rate of less than 100% in the table. As detailed under Methods, by changing the threshold for calling, a modest reduction in the call rate to 94.9% yields a 99.6% concordance rate.

**Table 3:** Results from Dynamic-variable LDA

			*High call rate*		*Lower call rate*	
			
*Training set*	*Test set*	*No. of SNPs*	*Call rate*	*Concordance rate*	*Call rate*	*Concordance rate*
Coriell	SIRS	96	99.6%	98.9%	94.9%	99.6%
SIRS	Coriell	102	99.9%	99.3%	95.6%	99.8%
Coriell	CV	96	100.0%	98.7%	94.2%	99.2%
SIRS	CV	102	99.9%	99.3%	96.0%	99.8%

We also reverse the roles of the training and test data sets, leading to the second row of Table 3. The results are stronger in terms of the number of SNPs called, call rate and concordance rate, because in this second analysis a much larger set of data is used for training the models.

Row 3 of Table 3 reports the results from applying the method of cross validation (CV) [14] to the Coriell data set. Here, each sample is removed in turn from the data, and its genotype is predicted based on retraining the four classifiers using *only the remaining data*. The results are similar to those in row 1. For the SIRS data, row 4 reports analogous cross validation performance estimates, and there is very close agreement with row 2.

### Simple LDA Based Genotyping Model

For comparison, for each SNP we use the training data to build a single LDA classification model using all eight variables available in Table 2. For each SNP, simple LDA applied to the training data assigns weights to the eight variables and these weights are constant for every test sample. Thus, this more standard modeling approach does not allocate weights dynamically. The same comment applies to MACGT from our research group [15], which also requires greater levels of manual inspection of the APEX data.

The results from simple linear discriminant analysis are given in Table 4. In row 1 the concordance rate for the SIRS test set is 97.3%, which might be considered good for other applications but for clinical purposes a much smaller concordance error is desirable. Modifying the calling threshold makes negligible difference to the concordance rate. Reversing the training and test data shows an even worse outcome: (1) again changing the threshold value does not control the call rate and (2) the concordance rate deteriorates drammatically. Therefore the performance is not competitive against dynamic-variable LDA.

**Table 4:** Results from Simple LDA

			*High call rate*		*Lower call rate*	
			
*Training set*	*Test set*	*No. of SNPs*	*Call rate*	*Concordance rate*	*Call rate*	*Concordance rate*
Coriell	SIRS	96	99.4%	97.3%	98.1%	97.3%
SIRS	Coriell	102	99.5%	93.0%	99.5%	93.0%
Coriell	CV	96	99.8%	98.4%	99.7%	98.5%
SIRS	CV	102	99.4%	99.5%	98.9%	99.6%

As shown in rows 3 and 4 of Table 4, the performance of simple linear discriminant analysis is better when measured by cross validation, particularly when predicting the SIRS data. It seems that the method is not robust to using samples from different sources for training and testing.

### Discussion

We also tried classifiers based on different sets of variables. For example, we built an ASO classifier using the variables ASO.XL, ASO.YL, ASO.XR and ASO.YR and an APEX classifier using the variables APEX.XL, APEX.YL, APEX.XR and APEX.YR. The calls from the two classifiers were then combined using the dynamic variable methodology. Little improvement in concordance rate was found relative to eight-variable simple LDA. Similar results were obtained when combining left and right classifiers, based on the left variables (ASO.XL, ASO.YL, APEX.XL and APEX.YL) and the right variables (ASO.XR, ASO.YR, APEX.XR and APEX.YR), respectively.

## Conclusion

We have developed a robust automated genotype calling method based on an ASO and APEX microarray platform. Multiple, qualitatively different probes provide redundancies in the event that a probe does not provide a reliable signal. The dynamic-variable calling algorithm respects these redundancies, building up an overall call from classifiers based on subsets of variables, with more weight given to seemingly more reliable classifiers. The weights change from one test sample to another; it is in this sense that the method is dynamic. Standard methods of variable selection (also called feature extraction) as described by, for example, Hand, Mannila, and Smyth [14] or Hastie, Tibshirani, and Friedman [16], would select or filter the variables and use the same set of reduced variables for every call. Such a strategy would be appropriate if the *same *probe sets are reliable from sample to sample.

For a call rate of approximately 95%, we were able to achieve a concordance rate of 99.6% in a large, independent test set of validated genotypes. The probe data for those samples/SNPs that are not automatically called would be manually inspected within SNP Chart; unlike 100% manual inspection, this does not impose an unreasonable time burden. The method of combining classifiers is not specific to linear discriminant analysis; other statistical classifiers could be used. Similarly, the method could be applied to other microarray platforms with complex redundancies.

## Methods

### LDA

Linear discriminant analysis (LDA), due to Fisher [13], is one of the oldest methods of discrimination between classes or classification. It is described in virtually every text book that includes classification (e.g., Hastie, Tibshirani, and Friedman) [16].

LDA is applied to each SNP separately. It is assumed that the variables (probe signals) used to classify have a multivariate normal distribution, with a within-class covariance matrix that is common to all classes (the genotypes and a negative control class) but within-class mean vectors that vary from one class to another. These quantities are estimated from the Coriell training data. For any test sample, the values of the same variables lead to posterior probabilities for the various classes. The genotype called is the class with the highest posterior probability. The method also requires the prior probabilities of belonging to the various classes. We assume priors based on observed frequencies in the training data. This basic LDA methodology is common to all the strategies we use.

### Simple LDA

In *Simple LDA *we train a single LDA genotyping model using the logarithms of all eight variables described in Table 2. Among the validated genotypes of the 32 Coriell samples, there are some cases where the exact genotype is unknown, denoted by NN (non-call). The three negative controls added to the Coriell data are also treated as NN as well. Thus, for each SNP there may be up to four classes present in the training data, corresponding to the three candidate genotypes and NN. Thus, LDA may call NN. The call rate is the proportion of calls that are not NN.

### Dynamic-variable LDA

For each SNP we apply LDA to each pair of variables in Table 5. For example, the classifier ASO.L is based on the left ASO variables, log(ASO.XL) and log(ASO.YL). For generic alleles X and Y, the classes are XX, XY, YY, and NN (if all are present). Table 6 sets out the notation for the Bayesian posterior probabilities for the possible classes from each of the four possible classifiers. For example, PXX(ASO.L)
 MathType@MTEF@5@5@+=feaafiart1ev1aaatCvAUfKttLearuWrP9MDH5MBPbIqV92AaeXatLxBI9gBaebbnrfifHhDYfgasaacH8akY=wiFfYdH8Gipec8Eeeu0xXdbba9frFj0=OqFfea0dXdd9vqai=hGuQ8kuc9pgc9s8qqaq=dirpe0xb9q8qiLsFr0=vr0=vr0dc8meaabaqaciaacaGaaeqabaqabeGadaaakeaacqWGqbaudaqhaaWcbaGaeeiwaGLaeeiwaGfabaGaeiikaGIaeeyqaeKaee4uamLaee4ta8KaeiOla4IaeeitaWKaeiykaKcaaaaa@3780@ is the posterior probability for the XX genotype from the classifier, ASO.L. The posterior probabilities for the four classifiers are combined using an entropy weighting scheme. Entropy is a measure of uncertainty or dispersion of a random variable. Denote the four posterior probabilities from any classifier (*) in any row of Table 6 by Pc∗
 MathType@MTEF@5@5@+=feaafiart1ev1aaatCvAUfKttLearuWrP9MDH5MBPbIqV92AaeXatLxBI9gBaebbnrfifHhDYfgasaacH8akY=wiFfYdH8Gipec8Eeeu0xXdbba9frFj0=OqFfea0dXdd9vqai=hGuQ8kuc9pgc9s8qqaq=dirpe0xb9q8qiLsFr0=vr0=vr0dc8meaabaqaciaacaGaaeqabaqabeGadaaakeaacqWGqbaudaqhaaWcbaGaem4yamgabaGaey4fIOcaaaaa@3040@, where *c *indexes one of the classes (genotypes) in the set

**Table 5:** Applying LDA using four sets of classifiers

*Classifier*	*Variables*
ASO.L	log(ASO.XL), log(ASO.YL)
ASO.R	log(ASO.XR), log(ASO.YR)
APEX.L	log(APEX.XL), log(APEX.YL)
APEX.R	log(APEX.XR), log(APEX.YR)

**Table 6:** Posterior probabilities from four LDA classifiers

*Classifier/Class*	XX	XY	YY	NN
ASO.L	PXX(ASO.L) MathType@MTEF@5@5@+=feaafiart1ev1aaatCvAUfKttLearuWrP9MDH5MBPbIqV92AaeXatLxBI9gBaebbnrfifHhDYfgasaacH8akY=wiFfYdH8Gipec8Eeeu0xXdbba9frFj0=OqFfea0dXdd9vqai=hGuQ8kuc9pgc9s8qqaq=dirpe0xb9q8qiLsFr0=vr0=vr0dc8meaabaqaciaacaGaaeqabaqabeGadaaakeaacqWGqbaudaqhaaWcbaGaeeiwaGLaeeiwaGfabaGaeiikaGIaeeyqaeKaee4uamLaee4ta8KaeiOla4IaeeitaWKaeiykaKcaaaaa@3780@	PXY(ASO.L) MathType@MTEF@5@5@+=feaafiart1ev1aaatCvAUfKttLearuWrP9MDH5MBPbIqV92AaeXatLxBI9gBaebbnrfifHhDYfgasaacH8akY=wiFfYdH8Gipec8Eeeu0xXdbba9frFj0=OqFfea0dXdd9vqai=hGuQ8kuc9pgc9s8qqaq=dirpe0xb9q8qiLsFr0=vr0=vr0dc8meaabaqaciaacaGaaeqabaqabeGadaaakeaacqWGqbaudaqhaaWcbaGaeeiwaGLaeeywaKfabaGaeiikaGIaeeyqaeKaee4uamLaee4ta8KaeiOla4IaeeitaWKaeiykaKcaaaaa@3782@	PYY(ASO.L) MathType@MTEF@5@5@+=feaafiart1ev1aaatCvAUfKttLearuWrP9MDH5MBPbIqV92AaeXatLxBI9gBaebbnrfifHhDYfgasaacH8akY=wiFfYdH8Gipec8Eeeu0xXdbba9frFj0=OqFfea0dXdd9vqai=hGuQ8kuc9pgc9s8qqaq=dirpe0xb9q8qiLsFr0=vr0=vr0dc8meaabaqaciaacaGaaeqabaqabeGadaaakeaacqWGqbaudaqhaaWcbaGaeeywaKLaeeywaKfabaGaeiikaGIaeeyqaeKaee4uamLaee4ta8KaeiOla4IaeeitaWKaeiykaKcaaaaa@3784@	PNN(ASO.L) MathType@MTEF@5@5@+=feaafiart1ev1aaatCvAUfKttLearuWrP9MDH5MBPbIqV92AaeXatLxBI9gBaebbnrfifHhDYfgasaacH8akY=wiFfYdH8Gipec8Eeeu0xXdbba9frFj0=OqFfea0dXdd9vqai=hGuQ8kuc9pgc9s8qqaq=dirpe0xb9q8qiLsFr0=vr0=vr0dc8meaabaqaciaacaGaaeqabaqabeGadaaakeaacqWGqbaudaqhaaWcbaGaeeOta4KaeeOta4eabaGaeiikaGIaeeyqaeKaee4uamLaee4ta8KaeiOla4IaeeitaWKaeiykaKcaaaaa@3758@
ASO.R	PXX(ASO.R) MathType@MTEF@5@5@+=feaafiart1ev1aaatCvAUfKttLearuWrP9MDH5MBPbIqV92AaeXatLxBI9gBaebbnrfifHhDYfgasaacH8akY=wiFfYdH8Gipec8Eeeu0xXdbba9frFj0=OqFfea0dXdd9vqai=hGuQ8kuc9pgc9s8qqaq=dirpe0xb9q8qiLsFr0=vr0=vr0dc8meaabaqaciaacaGaaeqabaqabeGadaaakeaacqWGqbaudaqhaaWcbaGaeeiwaGLaeeiwaGfabaGaeiikaGIaeeyqaeKaee4uamLaee4ta8KaeiOla4IaeeOuaiLaeiykaKcaaaaa@378C@	PXY(ASO.R) MathType@MTEF@5@5@+=feaafiart1ev1aaatCvAUfKttLearuWrP9MDH5MBPbIqV92AaeXatLxBI9gBaebbnrfifHhDYfgasaacH8akY=wiFfYdH8Gipec8Eeeu0xXdbba9frFj0=OqFfea0dXdd9vqai=hGuQ8kuc9pgc9s8qqaq=dirpe0xb9q8qiLsFr0=vr0=vr0dc8meaabaqaciaacaGaaeqabaqabeGadaaakeaacqWGqbaudaqhaaWcbaGaeeiwaGLaeeywaKfabaGaeiikaGIaeeyqaeKaee4uamLaee4ta8KaeiOla4IaeeOuaiLaeiykaKcaaaaa@378E@	PYY(ASO.R) MathType@MTEF@5@5@+=feaafiart1ev1aaatCvAUfKttLearuWrP9MDH5MBPbIqV92AaeXatLxBI9gBaebbnrfifHhDYfgasaacH8akY=wiFfYdH8Gipec8Eeeu0xXdbba9frFj0=OqFfea0dXdd9vqai=hGuQ8kuc9pgc9s8qqaq=dirpe0xb9q8qiLsFr0=vr0=vr0dc8meaabaqaciaacaGaaeqabaqabeGadaaakeaacqWGqbaudaqhaaWcbaGaeeywaKLaeeywaKfabaGaeiikaGIaeeyqaeKaee4uamLaee4ta8KaeiOla4IaeeOuaiLaeiykaKcaaaaa@3790@	PNN(ASO.R) MathType@MTEF@5@5@+=feaafiart1ev1aaatCvAUfKttLearuWrP9MDH5MBPbIqV92AaeXatLxBI9gBaebbnrfifHhDYfgasaacH8akY=wiFfYdH8Gipec8Eeeu0xXdbba9frFj0=OqFfea0dXdd9vqai=hGuQ8kuc9pgc9s8qqaq=dirpe0xb9q8qiLsFr0=vr0=vr0dc8meaabaqaciaacaGaaeqabaqabeGadaaakeaacqWGqbaudaqhaaWcbaGaeeOta4KaeeOta4eabaGaeiikaGIaeeyqaeKaee4uamLaee4ta8KaeiOla4IaeeOuaiLaeiykaKcaaaaa@3764@
APEX.L	PXX(APEX.L) MathType@MTEF@5@5@+=feaafiart1ev1aaatCvAUfKttLearuWrP9MDH5MBPbIqV92AaeXatLxBI9gBaebbnrfifHhDYfgasaacH8akY=wiFfYdH8Gipec8Eeeu0xXdbba9frFj0=OqFfea0dXdd9vqai=hGuQ8kuc9pgc9s8qqaq=dirpe0xb9q8qiLsFr0=vr0=vr0dc8meaabaqaciaacaGaaeqabaqabeGadaaakeaacqWGqbaudaqhaaWcbaGaeeiwaGLaeeiwaGfabaGaeiikaGIaeeyqaeKaeeiuaaLaeeyrauKaeeiwaGLaeiOla4IaeeitaWKaeiykaKcaaaaa@389D@	PXY(APEX.L) MathType@MTEF@5@5@+=feaafiart1ev1aaatCvAUfKttLearuWrP9MDH5MBPbIqV92AaeXatLxBI9gBaebbnrfifHhDYfgasaacH8akY=wiFfYdH8Gipec8Eeeu0xXdbba9frFj0=OqFfea0dXdd9vqai=hGuQ8kuc9pgc9s8qqaq=dirpe0xb9q8qiLsFr0=vr0=vr0dc8meaabaqaciaacaGaaeqabaqabeGadaaakeaacqWGqbaudaqhaaWcbaGaeeiwaGLaeeywaKfabaGaeiikaGIaeeyqaeKaeeiuaaLaeeyrauKaeeiwaGLaeiOla4IaeeitaWKaeiykaKcaaaaa@389F@	PYY(APEX.L) MathType@MTEF@5@5@+=feaafiart1ev1aaatCvAUfKttLearuWrP9MDH5MBPbIqV92AaeXatLxBI9gBaebbnrfifHhDYfgasaacH8akY=wiFfYdH8Gipec8Eeeu0xXdbba9frFj0=OqFfea0dXdd9vqai=hGuQ8kuc9pgc9s8qqaq=dirpe0xb9q8qiLsFr0=vr0=vr0dc8meaabaqaciaacaGaaeqabaqabeGadaaakeaacqWGqbaudaqhaaWcbaGaeeywaKLaeeywaKfabaGaeiikaGIaeeyqaeKaeeiuaaLaeeyrauKaeeiwaGLaeiOla4IaeeitaWKaeiykaKcaaaaa@38A1@	PNN(APEX.L) MathType@MTEF@5@5@+=feaafiart1ev1aaatCvAUfKttLearuWrP9MDH5MBPbIqV92AaeXatLxBI9gBaebbnrfifHhDYfgasaacH8akY=wiFfYdH8Gipec8Eeeu0xXdbba9frFj0=OqFfea0dXdd9vqai=hGuQ8kuc9pgc9s8qqaq=dirpe0xb9q8qiLsFr0=vr0=vr0dc8meaabaqaciaacaGaaeqabaqabeGadaaakeaacqWGqbaudaqhaaWcbaGaeeOta4KaeeOta4eabaGaeiikaGIaeeyqaeKaeeiuaaLaeeyrauKaeeiwaGLaeiOla4IaeeitaWKaeiykaKcaaaaa@3875@
APEX.R	PXX(APEX.R) MathType@MTEF@5@5@+=feaafiart1ev1aaatCvAUfKttLearuWrP9MDH5MBPbIqV92AaeXatLxBI9gBaebbnrfifHhDYfgasaacH8akY=wiFfYdH8Gipec8Eeeu0xXdbba9frFj0=OqFfea0dXdd9vqai=hGuQ8kuc9pgc9s8qqaq=dirpe0xb9q8qiLsFr0=vr0=vr0dc8meaabaqaciaacaGaaeqabaqabeGadaaakeaacqWGqbaudaqhaaWcbaGaeeiwaGLaeeiwaGfabaGaeiikaGIaeeyqaeKaeeiuaaLaeeyrauKaeeiwaGLaeiOla4IaeeOuaiLaeiykaKcaaaaa@38A9@	PXY(APEX.R) MathType@MTEF@5@5@+=feaafiart1ev1aaatCvAUfKttLearuWrP9MDH5MBPbIqV92AaeXatLxBI9gBaebbnrfifHhDYfgasaacH8akY=wiFfYdH8Gipec8Eeeu0xXdbba9frFj0=OqFfea0dXdd9vqai=hGuQ8kuc9pgc9s8qqaq=dirpe0xb9q8qiLsFr0=vr0=vr0dc8meaabaqaciaacaGaaeqabaqabeGadaaakeaacqWGqbaudaqhaaWcbaGaeeiwaGLaeeywaKfabaGaeiikaGIaeeyqaeKaeeiuaaLaeeyrauKaeeiwaGLaeiOla4IaeeOuaiLaeiykaKcaaaaa@38AB@	PYY(APEX.R) MathType@MTEF@5@5@+=feaafiart1ev1aaatCvAUfKttLearuWrP9MDH5MBPbIqV92AaeXatLxBI9gBaebbnrfifHhDYfgasaacH8akY=wiFfYdH8Gipec8Eeeu0xXdbba9frFj0=OqFfea0dXdd9vqai=hGuQ8kuc9pgc9s8qqaq=dirpe0xb9q8qiLsFr0=vr0=vr0dc8meaabaqaciaacaGaaeqabaqabeGadaaakeaacqWGqbaudaqhaaWcbaGaeeywaKLaeeywaKfabaGaeiikaGIaeeyqaeKaeeiuaaLaeeyrauKaeeiwaGLaeiOla4IaeeOuaiLaeiykaKcaaaaa@38AD@	PNN(APEX.R) MathType@MTEF@5@5@+=feaafiart1ev1aaatCvAUfKttLearuWrP9MDH5MBPbIqV92AaeXatLxBI9gBaebbnrfifHhDYfgasaacH8akY=wiFfYdH8Gipec8Eeeu0xXdbba9frFj0=OqFfea0dXdd9vqai=hGuQ8kuc9pgc9s8qqaq=dirpe0xb9q8qiLsFr0=vr0=vr0dc8meaabaqaciaacaGaaeqabaqabeGadaaakeaacqWGqbaudaqhaaWcbaGaeeOta4KaeeOta4eabaGaeiikaGIaeeyqaeKaeeiuaaLaeeyrauKaeeiwaGLaeiOla4IaeeOuaiLaeiykaKcaaaaa@3881@

*C *= {XX, XY, YY, NN}.

Then the entropy for this probability distribution over classes is defined to be

−∑c∈CPc∗log⁡(Pc∗).
 MathType@MTEF@5@5@+=feaafiart1ev1aaatCvAUfKttLearuWrP9MDH5MBPbIqV92AaeXatLxBI9gBaebbnrfifHhDYfgasaacH8akY=wiFfYdH8Gipec8Eeeu0xXdbba9frFj0=OqFfea0dXdd9vqai=hGuQ8kuc9pgc9s8qqaq=dirpe0xb9q8qiLsFr0=vr0=vr0dc8meaabaqaciaacaGaaeqabaqabeGadaaakeaacqGHsisldaaeqbqaaiabdcfaqnaaDaaaleaacqWGJbWyaeaacqGHxiIkaaaabaGaem4yamMaeyicI4Saem4qameabeqdcqGHris5aOGagiiBaWMaei4Ba8Maei4zaCMaeiikaGIaemiuaa1aa0baaSqaaiabdogaJbqaaiabgEHiQaaakiabcMcaPiabc6caUaaa@4183@

Entropy or uncertainty is maximized when all the Pc∗
 MathType@MTEF@5@5@+=feaafiart1ev1aaatCvAUfKttLearuWrP9MDH5MBPbIqV92AaeXatLxBI9gBaebbnrfifHhDYfgasaacH8akY=wiFfYdH8Gipec8Eeeu0xXdbba9frFj0=OqFfea0dXdd9vqai=hGuQ8kuc9pgc9s8qqaq=dirpe0xb9q8qiLsFr0=vr0=vr0dc8meaabaqaciaacaGaaeqabaqabeGadaaakeaacqWGqbaudaqhaaWcbaGaem4yamgabaGaey4fIOcaaaaa@3040@'s are equal and minimized (taking the value 0) when one of the Pc∗
 MathType@MTEF@5@5@+=feaafiart1ev1aaatCvAUfKttLearuWrP9MDH5MBPbIqV92AaeXatLxBI9gBaebbnrfifHhDYfgasaacH8akY=wiFfYdH8Gipec8Eeeu0xXdbba9frFj0=OqFfea0dXdd9vqai=hGuQ8kuc9pgc9s8qqaq=dirpe0xb9q8qiLsFr0=vr0=vr0dc8meaabaqaciaacaGaaeqabaqabeGadaaakeaacqWGqbaudaqhaaWcbaGaem4yamgabaGaey4fIOcaaaaa@3040@'s is 1 and the others are zero.

Entropy is computed for each of the four classifiers in Table 6. We will be giving more weight to a classifier with *less *entropy (uncertainty). Thus, we define for the ASO.L classifier in row 1 of the table, for example,

EASO.L=−log⁡(14)−[−∑c∈CPc(ASO.L)log⁡(Pc(ASO.L))],
 MathType@MTEF@5@5@+=feaafiart1ev1aaatCvAUfKttLearuWrP9MDH5MBPbIqV92AaeXatLxBI9gBaebbnrfifHhDYfgasaacH8akY=wiFfYdH8Gipec8Eeeu0xXdbba9frFj0=OqFfea0dXdd9vqai=hGuQ8kuc9pgc9s8qqaq=dirpe0xb9q8qiLsFr0=vr0=vr0dc8meaabaqaciaacaGaaeqabaqabeGadaaakeaacqWGfbqrdaWgaaWcbaGaeeyqaeKaee4uamLaee4ta8KaeiOla4IaeeitaWeabeaakiabg2da9iabgkHiTiGbcYgaSjabc+gaVjabcEgaNjabcIcaOmaalaaabaGaeGymaedabaGaeGinaqdaaiabcMcaPiabgkHiTiabcUfaBjabgkHiTmaaqafabaGaemiuaa1aa0baaSqaaiabdogaJbqaaiabcIcaOiabbgeabjabbofatjabb+eapjabc6caUiabbYeamjabcMcaPaaaaeaacqWGJbWycqGHiiIZcqWGdbWqaeqaniabggHiLdGccyGGSbaBcqGGVbWBcqGGNbWzcqGGOaakcqWGqbaudaqhaaWcbaGaem4yamgabaGaeiikaGIaeeyqaeKaee4uamLaee4ta8KaeiOla4IaeeitaWKaeiykaKcaaOGaeiykaKIaeiyxa0LaeiilaWcaaa@618E@

which is a quantity which is large if ASO.L's entropy is small compared to the maximum possible entropy. Analogous quantities are computed for *E*_ASO.R_, *E*_APEX.L_, and *E*_APEX.R _in Table 6. The weights for the four classifiers are obtained by normalizing them so that they sum to 1, i.e.,

WASO.L=EASO.LEASO.L+EASO.R+EAPEX.L+EAPEX.R,
 MathType@MTEF@5@5@+=feaafiart1ev1aaatCvAUfKttLearuWrP9MDH5MBPbIqV92AaeXatLxBI9gBaebbnrfifHhDYfgasaacH8akY=wiFfYdH8Gipec8Eeeu0xXdbba9frFj0=OqFfea0dXdd9vqai=hGuQ8kuc9pgc9s8qqaq=dirpe0xb9q8qiLsFr0=vr0=vr0dc8meaabaqaciaacaGaaeqabaqabeGadaaakeaacqWGxbWvdaWgaaWcbaGaeeyqaeKaee4uamLaee4ta8KaeeOla4IaeeitaWeabeaakiabg2da9maalaaabaGaemyrau0aaSbaaSqaaiabbgeabjabbofatjabb+eapjabb6caUiabbYeambqabaaakeaacqWGfbqrdaWgaaWcbaGaeeyqaeKaee4uamLaee4ta8KaeeOla4IaeeitaWeabeaakiabgUcaRiabdweafnaaBaaaleaacqqGbbqqcqqGtbWucqqGpbWtcqqGUaGlcqqGsbGuaeqaaOGaey4kaSIaemyrau0aaSbaaSqaaiabbgeabjabbcfaqjabbweafjabbIfayjabb6caUiabbYeambqabaGccqGHRaWkcqWGfbqrdaWgaaWcbaGaeeyqaeKaeeiuaaLaeeyrauKaeeiwaGLaeeOla4IaeeOuaifabeaaaaGccqGGSaalaaa@5BA2@

with analogous computations for *W*_ASO.R_, *W*_APEX.L_, and *W*_APEX.R_. Note that the probabilities in Table 6 and hence the weights will vary from one test sample to another.

The weights for the four classifiers are applied to the posterior probabilities for each class (column) in Table 6 to obtain the final class posterior probabilities. For example, the final probability for XX is

PXX=WASO.LPXX(ASO.L)+WASO.RPXX(ASO.R)+WAPEX.LPXX(APEX.L)+WAPEX.RPXX(APEX.R),
 MathType@MTEF@5@5@+=feaafiart1ev1aaatCvAUfKttLearuWrP9MDH5MBPbIqV92AaeXatLxBI9gBaebbnrfifHhDYfgasaacH8akY=wiFfYdH8Gipec8Eeeu0xXdbba9frFj0=OqFfea0dXdd9vqai=hGuQ8kuc9pgc9s8qqaq=dirpe0xb9q8qiLsFr0=vr0=vr0dc8meaabaqaciaacaGaaeqabaqabeGadaaakeaacqWGqbaudaWgaaWcbaGaeeiwaGLaeeiwaGfabeaakiabg2da9iabdEfaxnaaBaaaleaacqqGbbqqcqqGtbWucqqGpbWtcqqGUaGlcqqGmbataeqaaOGaemiuaa1aa0baaSqaaiabbIfayjabbIfaybqaaiabcIcaOiabbgeabjabbofatjabb+eapjabb6caUiabbYeamjabcMcaPaaakiabgUcaRiabdEfaxnaaBaaaleaacqqGbbqqcqqGtbWucqqGpbWtcqqGUaGlcqqGsbGuaeqaaOGaemiuaa1aa0baaSqaaiabbIfayjabbIfaybqaaiabcIcaOiabbgeabjabbofatjabb+eapjabb6caUiabbkfasjabcMcaPaaakiabgUcaRiabdEfaxnaaBaaaleaacqqGbbqqcqqGqbaucqqGfbqrcqqGybawcqqGUaGlcqqGmbataeqaaOGaemiuaa1aa0baaSqaaiabbIfayjabbIfaybqaaiabcIcaOiabbgeabjabbcfaqjabbweafjabbIfayjabb6caUiabbYeamjabcMcaPaaakiabgUcaRiabdEfaxnaaBaaaleaacqqGbbqqcqqGqbaucqqGfbqrcqqGybawcqqGUaGlcqqGsbGuaeqaaOGaemiuaa1aa0baaSqaaiabbIfayjabbIfaybqaaiabcIcaOiabbgeabjabbcfaqjabbweafjabbIfayjabb6caUiabbkfasjabcMcaPaaakiabcYcaSaaa@8045@

with similar calculations for XY, YY, and NN. A sample is assigned to the class with maximum weighted probability.

To increase the concordance with the validated test samples (at the expense of reducing the call rate), a call is made if and only if the maximum probability across the classes exceeds a threshold. For instance, the results in the last two columns of the first row of Table 3 are obtained by requiring the maximum probability to be at least 0.6 for a call.

#### Example corresponding to SNP rs1003399 and sample Coriell NA17111

Figure 3 relates to SNP rs1003399 and the point labeled 11 is Coriell sample NA17111. To check how dynamic-variable LDA works for a sample with complex redundancy, we predict the genotype of that sample based on the remaining 31 Coriell samples plus three negative PCR controls. Underlying calculations for both dynamic-variable LDA and simple LDA are shown here.

The posterior probabilities from dynamic-variable LDA corresponding to Table 6 but specific to this example are given in Table 7. The final posterior probabilities from Dynamic-variable LDA and Simple LDA are given in Table 8. So from Table 8, it is clear that the sample Coriell NA17111 (with validated genotype GG) is correctly classified only by dynamic-variable LDA with confidence measure .75, but simple LDA fails to do so. Moreover simple LDA wrongly classifies the sample as CG with high confidence score (posterior probability 1.000).

**Table 7:** Posterior probabilities from Table 6 for SNP rs1003399 and target sample Coriell NA17111

*Classifier/Class*	CC	CG	GG	NN
ASO.L	<0.001	0.001	0.999	<0.001
ASO.R	<0.001	0.003	0.997	<0.001
APEX.L	<0.001	1.000	<0.001	<0.001
APEX.R	<0.001	0.005	0.995	<0.001

**Table 8:** Resultant posterior probabilities from two methods

*Classes/Methods*	Dynamic-variable LDA	Simple LDA
CC	<0.001	<0.001
CG	0.253	1.000
GG	0.746	<0.001
NN	<0.001	<0.001

## Competing interests

The author(s) declare that they have no competing interests.

## Authors' contributions

MP designed and developed the algorithms, performed the statistical analysis of the data sets and drafted the manuscript; all the authors contributed to the writing of the final version. WJW and RHZ supervised in developing the algorithms. SJT designed the APEX microarray chip and provided the data sets. All authors read and approved the final manuscript.
